# Management of childhood granulomatous periorificial dermatitis with clarithromycin: A retrospective cohort study

**DOI:** 10.1016/j.jdin.2025.08.005

**Published:** 2025-08-29

**Authors:** Yizhu Xiao, Jingyi He, Anwei Chen, Xiao Fang, Ping Tang, Juan Xiang

**Affiliations:** Department of Dermatology, Children’s Hospital of Chongqing Medical University, National Clinical Research Center for Child Health and Disorders, Ministry of Education Key Laboratory of Child Development and Disorders, China International Science and Technology Cooperation Base of Child Development and Critical Disorders, Chongqing Key Laboratory of Child Rare Diseases in Infection and Immunity, Chongqing, China

**Keywords:** childhood granulomatous periorificial dermatitis, clarithromycin, scarring, systemic intervention

## Abstract

**Background:**

Childhood granulomatous periorificial dermatitis incidence is increasing, yet topical therapies remain suboptimal with chronicity and scarring risks in severe cases. Systemic alternatives are urgently needed. Although oral clarithromycin shows promise, its efficacy/safety evidence in pediatric populations lacks robustness.

**Objective:**

This study evaluated the effect of clarithromycin on a pediatric group with granulomatous periorificial dermatitis.

**Methods:**

This retrospective study analyzed 43 patients treated with oral clarithromycin, with outcomes including treatment efficacy, adverse event incidence, and scar incidence.

**Results:**

All patients completed clarithromycin therapy (median 10 weeks; 2-26). At 6 months, 95.4% (41/43) cleared, with 2.3% each recurrence and nonresponse. Treatment duration correlated with both baseline severity (*r* = 0.592, *P* < .01) and disease duration (*r* = 0.590, *P* < .01). Scarring (14.6%) occurred only in moderate-to-severe cases, which showed greater severity (*P* = .039) and longer courses (*P* = .028). No severe adverse events occurred.

**Limitations:**

This is a retrospective study without case controls and is subject to interviewer and memory bias.

**Conclusion:**

For moderate-to-severe or stubborn cases, timely evaluation and tailored systemic interventions are critical to mitigate scarring risks. Clarithromycin is an effective and tolerated therapy in children with moderate-to-severe and refractory granulomatous periorificial dermatitis.

**Capsule Summary**

• Childhood granulomatous periorificial dermatitis carries underrecognized scarring risks and poses a persistent therapeutic challenge. Severe or refractory cases require prompt systemic interventions to mitigate scarring risks.
• Clarithromycin serves as a well-tolerated therapeutic alternative in the pediatric population.

## Introduction

Childhood granulomatous periorificial dermatitis (CGPD) is a distinctive granulomatous variant of periorificial dermatitis (POD) affecting prepubertal children. Typical clinical features include flesh-colored papules around the orifices area with vermillion-spared and noncaseating perifollicular granulomas infiltrate on examination of a biopsy specimen. Perioral dermatitis is a common cutaneous inflammation and presents in middle-aged women, characterized by acneiform facial erythematous papulopustular eruptions with an eczematous appearance, lacking granulomatous inflammation.^1^ Although primarily localized to facial orifices, accumulating case reports describe atypical manifestations involving extrafacial sites, including the forehead, neck, and vulvar regions.^2^ Although population-level epidemiologic evidence remains unavailable, clinicians increasingly report pediatric patients presenting with this condition in recent year.^3^ Existing but underrecognized evidence from 2 clinical cases documents postinflammatory scarring crisis.^4^^,^^5^ Current therapeutic guidelines remain controversial, particularly in pediatric populations. Therapeutic decision-making is complicated by contraindications to tetracyclines—the first-line therapy in adult granulomatous POD—given their association with dental discoloration and skeletal toxicity in children under 8 years.^6^ This clinical dilemma has prompted exploration of alternative agents, with preliminary evidence suggesting that clarithromycin's dual anti-inflammatory and antimicrobial properties may offer a steroid-sparing therapeutic option.^7^^,^^8^

In this context, we conducted a retrospective cohort study to characterize the clinical spectrum and possible triggers and systematically evaluate the therapeutic efficacy and safety profile of oral clarithromycin in pediatric populations, aiming to expand treatment options for this understudied population.

## Methods

This retrospective study included patients with CGPD (aged ≥6 months to <18 years) identified using International Classification of Diseases, Tenth Revision code L71.8 at our pediatric dermatology center from January 2020 to November 2024, with approval from the Ethics Committee of Chongqing Medical University in accordance with the Declaration of Helsinki. Inclusion criteria were as follows:

1. Clinical diagnosis of CGPD: All included patients had a documented clinical diagnosis of CGPD established by a board-certified dermatologist during their initial or follow-up visits, as recorded in their medical charts. Cases explicitly documented in the charts as “CGPD,” “childhood granulomatous periorificial dermatitis,” or with a clear synonymous description were included. On the other hand, for cases where the clinical presentation was recorded as atypical, inclusion required additional documentation of histopathologic examination results that were consistent with CGPD. As part of the retrospective data abstraction process, 2 independent reviewers cross-verified the documented dermatologist diagnosis against predefined CGPD clinical criteria. Any discrepancies or uncertainties were resolved through discussion with a senior dermatologist.
2. Disease severity or duration: Patients with either documented moderate-to-severe severity (severity index ≥ 4) or disease duration ≥6 months. Disease severity for each patient was retrospectively assessed using a modified 7-point scale (Lee and Kim^9^). This assessment was performed solely based on the information available within the patient’s medical records by 2 independent, trained reviewers. The scale incorporated the following elements: erythema intensity, lesion morphology (papules, pustules, scaling), and distribution (facial/extrafacial). Severity scores were derived from detailed textual descriptions within the clinical notes from the relevant visits, as well as clinical photographs attached to the electronic chart review if available. Severity grades include mild (1-3), moderate (4-5), and severe (6-7).
3. Clarithromycin-based systemic therapy: Treatment duration and reason for discontinuation were extracted from prescription records and clinical progress notes. No minimum treatment duration threshold was imposed for inclusion, as early termination represented clinically relevant outcomes. Based on chart review, oral clarithromycin (10-15 mg/kg/d, max 500 mg) was discontinued following documented complete clinical resolution, with patients having been monitored every 2 to 4 weeks during therapy. The recorded regimen involved oral clarithromycin plus topical calcineurin inhibitors (TCIs) (specifically, 0.03% tacrolimus ointment for patients aged ≥2 years and 1% pimecrolimus cream for those <2 years), then followed by maintenance therapy with TCIs after clarithromycin withdrawal in a sequential approach. Treatment response was classified as follows: complete response (CR): total lesion clearance without recurrence; partial response: ≥1-grade improvement without full resolution; nonresponse (NR): <1-grade improvement after 12 weeks; and recurrence: lesion reappearance postclearance after withdrawal of oral clarithromycin for 8 weeks.

Data extracted from electronic medical records included diagnosis details, rash distribution, disease duration, triggering factors, prior treatments, treatment regimens, clinical outcomes (treatment response, adverse events, recurrence rate, scarring incidence), and demographic/family history.

### Statistical analyses

Continuous variables are expressed as mean ± SD or median (range) based on distribution normality, and categorical variables are presented as numbers and percentages. Spearman's rank correlation coefficient is used for analyzing correlations. Group comparisons were performed with the Mann-Whitney U test. Statistical significance was defined as a 2-tailed *P* value <0.05 for all analyses.

## Results

### Patients

The cohort comprised 43 children (M:F = 26:17; mean onset age 4.9 years, range: 1.3-11.9) with a median preconsultation disease duration of 9.5 months (1-28). Comorbidities included atopic dermatitis (4.7%, 2/43), allergic rhinitis (14%, 6/43), and asthma (11.6%, 5/43). Subgroup analysis by disease severity revealed mild case (7%, 3/43) with a median duration of 12 months (range: 12-28), moderate (41.9%, 18/43) with a median duration of 6.8 months (range: 1-17), and severe (51.1%, 22/43) with a median duration of 10.6 months (range: 1-26). Prior to clarithromycin therapy, 32.6% of patients were untreated, while the majority underwent systemic/topical interventions. Systemic macrolide pretreatment included azithromycin/erythromycin (*n* = 1 each) without improvement. Topical regimens comprised corticosteroids (30.2%), TCIs (27.9%), antibiotics (18.6%), traditional herbal preparations (14%), and antifungals (4.7%) (Table I).

**Table I:** Patient features in 43 children with childhood granulomatous periorificial dermatitis

Characteristic	Value
Age, y	
Average, (range)	4.9 (1.3-11.9)
Gender, *n* (%)	
Male	26 (60.5)
Female	17 (39.5)
Severity, *n* (%)	
Mild	3 (7)
Moderate	18 (41.9)
Severe	22 (51.1)
Duration of disease, mo (range)	9.5 (1-28)
Mild	13 (12-28)
Moderate	6.8 (1-17)
Severe	10.6 (1-26)
Types of eruption, *n* (%)	
Erythema	43 (100)
Papules	40 (93)
Pustules	16 (37.2)
Desquamation	9 (20.9)
Location of lesions, *n* (%)	
Perioral	43 (100)
Perinasal	25 (58.1)
Periocular	18 (41.8)
All 3 orifices	14 (32.6)
Extrafacial regions	5 (11.6)
Cheek	1 (2.3)
Neck	2 (4.7)
Vulva	2 (4.7)
Previous treatments, *n* (%)	
Systematic antibiotics∗	2 (4.7)
Topical corticosteroids	13 (30.2)
TCIs	12 (27.9)
Topical antibiotics	8 (18.6)
Traditional herb cream	6 (14)
Topical antifungals	2 (4.7)
Personal atopy history, *n* (%)	
Atopic dermatitis	2 (4.7)
Allergic rhinitis	6 (14)
Asthma	5 (11.6)
Possible triggers, *n* (%)	
Inhaled corticosteroids	3 (7)
Topical corticosteroids	1 (2.3)
Fluoride toothpaste	1 (2.3)

*TCI*, Topical calcineurin inhibitor.

∗ Systemic antibiotics included azithromycin (1 patient), erythromycin (1 patient).

### Disease presentation and characteristics

**Fig 1:**
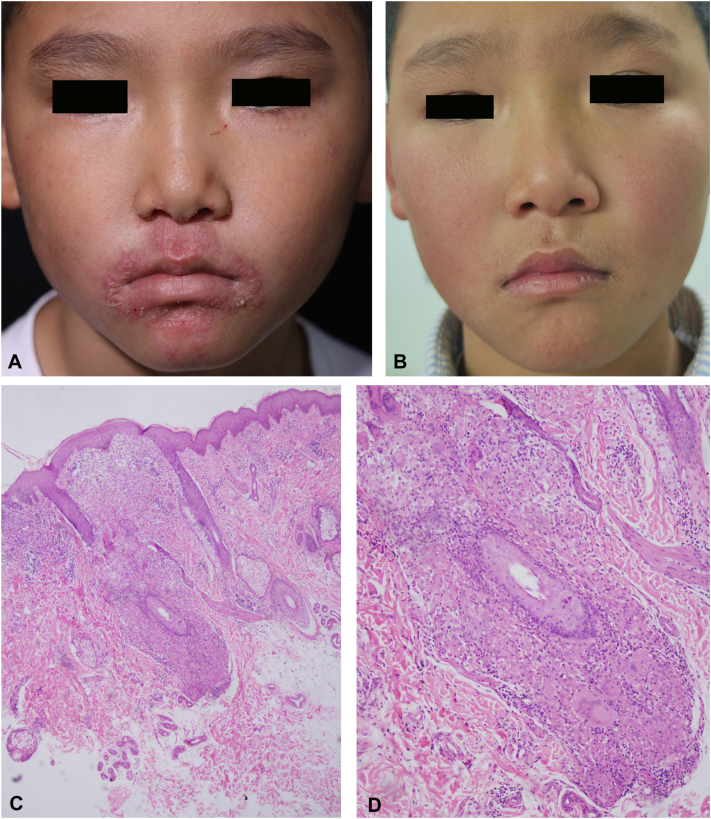
Clinical features, histologic manifestations, and therapeutic response to clarithromycin in childhood granulomatous periorificial dermatitis (CGPD). **(A)** CGPD. An 8-year-old boy presented with multiple, pinhead-sized, pinkish papules and marked erythema. The lesions were grouped predominantly around the mouth, eyes, and nose crease. **(B)** CGPD. Following 6 months of clarithromycin treatment, the rashes had completely subsided and superficial dotted, and linear atrophic scars were visible around the mouth. **(C)** CGPD. Hematoxylin-eosin staining shows granulomatous infiltrates located intradermally and around the hair follicles. (HE, ×40). **(D)** CGPD. At high magnification, perifollicular noncaseating granulomas with multinucleated giant cells are observed. (HE, ×200).

**Fig 2:**
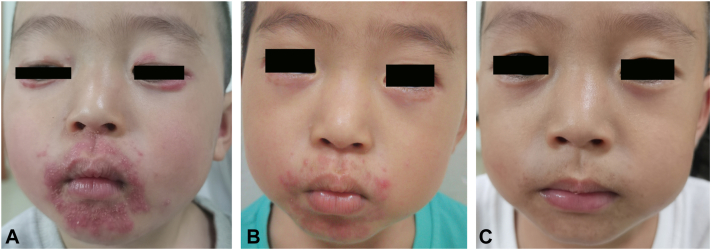
Childhood granulomatous periorificial dermatitis (CGPD). A 3-year and 3-month-old boy with granulomatous periorificial dermatitis before and after 4 months of oral clarithromycin therapy. **(A)** CGPD. Prior to treatment, persistent erythema and millet-sized papules with scattered pustules and crusting involved the perioral, perinasal, and periocular regions. **(B)** CGPD. At the first follow-up after 3 weeks of treatment, a significant reduction and regression of the erythema, papules, and papulopustules around the orifices were noted. **(C)** CGPD. Following 4 months of oral clarithromycin, the skin lesions totally disappeared, with residual pigmentation and pinpoint superficial scarring.

**Fig 3:**
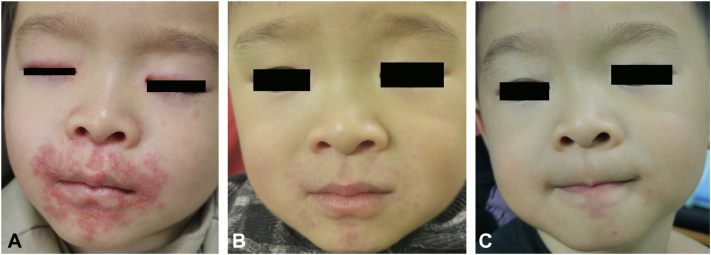
Childhood granulomatous periorificial dermatitis (CGPD). Complete response without sequela achieved after 9-week oral clarithromycin. **(A)** CGPD. A 2-year and 9-month-old child with a 4-month history of nonpruritic skin eruption on the face. Prominent erythema and multiple pinpoint yellowish papulopustules in the orifice. **(B)** CGPD. The eruptions were markedly ameliorated by 4 weeks of treatment. **(C)** CGPD. At the 6-month follow-up after medication withdrawal at 9 weeks, the rashes remained cleared without recurrence.

All 43 patients exhibited perioral involvement. The perinasal and periorbital regions were affected in 58.1% and 41.8% of cases, respectively, with concurrent involvement of all 3 facial regions (perioral-nasal-orbital) observed in 32.6%. Extraorificial involvement occurred in 11.6%, including vulvar (4.7%, 2/43), neck (4.7%, 2/43), and cheek (2.3%, 1/43) regions. Primary lesion morphology included erythematous papules (100%), with pustules present in 37.2% and concurrent desquamation in 20.9% (Fig 1, Fig 2, Fig 3). Histopathologic analysis of 7 biopsied cases consistently demonstrated nonnecrotizing granulomas in the superficial to mid dermis, especially around the perifollicular area, accompanied by lymphocytic infiltrates and multinucleated giant cells (Fig 1, *C* and *D*). Prior corticosteroid exposure (topical/inhaled) was documented in 9.3% of patients. One patient reported switching to fluoride-containing toothpaste preceding symptom onset.

### Treatment response

All patients received oral clarithromycin (10-15 mg/kg/d; maximum 500 mg/d) and were monitored at regular intervals of 2 to 4 weeks during systemic therapy (Fig 1, Fig 2, Fig 3). The median treatment duration was 10 weeks (range: 2-26), stratified as follows: ≤4 weeks (16.3%), ≤8 weeks (32.6%),≤12 weeks (20.9%), ≤24 weeks (27.9%), and >24 weeks (2.3%). Therapeutic outcomes showed 69.8% CR and 25.5% partial response at 3 months, with 1 nonresponse and 1 recurrence. Six-month CR rates rose to 95.4%, retaining 1 nonresponse and 1 relapse. Residual superficial scars occurred in 14.6% (6/41) of patients with CR: 5 were boys (4 cases were severe, 1 was moderate), while 1 was a girl with a severe condition (Figs 1 and 2, Table II). Treatment duration showed significant positive correlations with disease course (*r* = 0.592, *P* < .01) and baseline severity (*r* = 0.590, *P* < .01). Scar-associated cases demonstrated prolonged disease courses (*P* = .0283) and increased severity (*P* = .039) without treatment duration differences (*P* = .128).

**Table II:** Treatment, disease severity, and outcomes in 43 patients with childhood granulomatous periorificial dermatitis

Disease severity	Treatment period, W (R)	Treatment response, *n* (%)								Pitted scar *n* (%)
		12 wk				24 wk				
Totals	10 (2-26)	CR	PR	NR	Re	CR	PR	NR	Re	6 (14.6)
		30 (69.8)	11 (25.5)	1 (2.3)	1 (2.3)	41 (95.4)	0 (0)	1 (2.3)	1 (2.3)	
Mild	7.7 (2-18)	2 (4.7)	1 (2.3)	0 (0)	0 (0)	3 (6.9)	0 (0)	0 (0)	0 (0)	0 (0)
Moderate	7.2 (2-19)	13 (30.2)	4 (9.3)	0 (0)	1 (2.3)	18 (41.9)	0 (0)	0 (0)	0 (0)	1 (2.4)
Severe	12.4 (5-26)	15 (34.9)	6 (13.9)	1 (2.3)	0 (0)	20 (46.6)	0 (0)	1 (2.3)	1 (2.3)	5 (12.2)

*CR*, Complete response; *NR*, nonresponse; *PR*, partial response; *R*, range; *Re*, recurrence; *W*, week.

### Adverse event

Three patients (7.0%, 3/43) experienced transient cutaneous exacerbations within the first week of treatment initiation, resolving spontaneously with continued therapy. Liver and kidney function monitoring was performed in 25.6% (11/43) of patients, with all results remaining within normal limits. Laboratory testing was declined by 16.3% (7/43) of caregivers due to the absence of overt adverse effects, while 58.1% lacked documented test results. No severe adverse events occurred. Mild nausea (2.3%) resolved spontaneously within 48 hours.

## Discussion

CGPD primarily affects prepubertal children, with no documented racial predilection.^10^ In this retrospective cohort, a mild male predominance was observed, although potential selection biases require consideration. The most frequent cutaneous manifestation involved the perioral regions, while extrafacial involvement occurred in 5 patients. Sterile yellowish-white pustules, although rarely reported in pediatric dermatoses,^11^ were identified in 37.2% of cases. Notably, 6 patients developed residual shallow scarring postresolution, challenging prior assertions of universally benign outcomes. Preliminary observations suggest correlations between scarring risk and both disease severity and prolonged duration (>6 months). These findings highlight the need for prognostic stratification and further research to identify risk factors or mechanisms.

The exact etiology of CGPD remains undetermined. Although topical corticosteroid misuse is recognized as the primary risk factor,^12^ the causal relationship between steroid exposure and disease pathogenesis requires further elucidation. Hogan et al^13^ demonstrated prior topical corticosteroid use in 85% of POD cases, a finding contrasting with our pediatric cohort study, where only 9.3% reported steroid exposure, although 30.2% exhibited topical steroid application for pretreatment. Although the specific role of corticosteroids in POD is uncertain, topical steroids might alter the microflora of hair follicles, creating a microflora imbalance, contributing to the widely recognized symptoms of POD.^14^ Notably, a recent case reported crisaborole, a nonsteroidal phosphodiesterase-4 inhibitor approved for atopic dermatitis, was connected to exacerbation of pediatric POD.^15^ In fact, emerging evidence implicates diverse environmental exposures in CGPD pathogenesis, with documented associations including fluoride-containing toothpaste, dental restorations, and sunscreen components.^16^

Topical therapies (metronidazole, calcineurin inhibitors) are empirically used for mild pediatric GPD, yet systematic review indicates inconclusive efficacy and substantial relapse risks postdiscontinuation.^5^^,^^17^ Severe chronic cases often necessitate systemic interventions; however, therapeutic decisions remain challenged by developmental contraindications and heterogeneous disease trajectories, with limited evidence to guide age-specific protocols. Tetracyclines are contraindicated in children under 12 years, whereas oral isotretinoin—although effective in refractory adult cases—remains restricted in children due to skeletal toxicity risks.^18^ Oral metronidazole and macrolides have been reported as effective alternatives in pediatric cases,^19^^,^^20^ and latest studies suggest that macrolides may offer faster treatment responses and shorter courses compared with metronidazole.^21^ However, gastrointestinal intolerance associated with both drugs frequently complicates pediatric administration. Existing efficacy data reveal 72% complete resolution with azithromycin-topical combination therapy (mean 2.7 months)^22^ versus 90.3% remission using roxithromycin monotherapy (12 weeks), with 6.5% 6-month recurrence.^23^ Clarithromycin therapy demonstrated 95.4% sustained lesion clearance at the 6-month follow-up, with a 2.3% recurrence rate. The optimal duration of oral antibiotic therapy for pediatric GPD remains undefined. Our cohort achieved partial improvement within 2 weeks, with a median treatment duration of 10 weeks (range: 2-26); extended therapy was associated with prolonged disease courses and higher baseline clinical severity. Conversely, early discontinuation increased relapse risk. The wide range of reported regimens (6 weeks to 8 months) in the literature demonstrates the chronic nature of CGPD and the lack of standardized end points.^4^^,^^7^^,^^8^^,^^19^^,^^24^ More clinical studies are needed to establish better treatment durations that effectively balance therapeutic efficacy with minimizing potential risks. Scarring was associated with prolonged disease courses but did not correlate with treatment duration, suggesting that early clinical assessment and optimized intervention are crucial to minimize scarring risks. Clarithromycin demonstrated excellent safety in CGPD treatment, with no serious adverse events and minimal gastrointestinal reactions. These findings confirm clarithromycin's efficacy in treatment-resistant CGPD and support its use as an effective option for obstinate pediatric cases, with good tolerability.

### Limitation

This study has limitations inherent to its retrospective design, including potential biases, lack of a control group, and small sample size, which may affect generalizability. All patients concurrently used TCIs, although their efficacy was limited in this refractory CGPD cohort. Additional constraints include variability in treatment durations and absence of standardized protocols, complicating the determination of optimal therapy. Future studies require longer-term follow-up, control of confounding factors, and prospective designs with larger cohorts to establish evidence-based pediatric granulomatous POD guidelines.

## Conclusion

For moderate-to-severe CGPD, early systemic intervention is critical to prevent scarring sequelae. Our data demonstrate that oral clarithromycin serves as an effective and well-tolerated therapeutic option, achieving rapid lesion resolution with sustained clearance through 6-month follow-up.

## Conflicts of interest

None disclosed.
